# Correction: IL-6 is one of the key factors in the formation of gut tissue resident memory T cells from Naïve T cells

**DOI:** 10.1371/journal.ppat.1014176

**Published:** 2026-04-28

**Authors:** Han G. Kim, Amanda Chan, Sinmanus Vimonpatranon, Alexandre Girard, Andrew Jiang, Samuel Wertz, Il-Young Hwang, John H. Kehrl, Hana Schmeisser, Madelyn M. Seemiller, Paolo Lusso, Dawei Huang, Danlan Wei, Livia R. Goes, Marcelo Soares, Elena Martinelli, James Arthos, Claudia Cicala

The third author’s name is spelled incorrectly. The correct name is: Sinmanus Vimonpatranon. The correct citation is: Kim HG, Chan A, Vimonpatranon S, Girard A, Jiang A, Wertz S, et al. (2026) IL-6 is one of the key factors in the formation of gut tissue resident memory T cells from Naïve T cells. PLoS Pathog 22(3): e1014052. https://doi.org/10.1371/journal.ppat.1014052.
