# Supplementary figures and images for: IL-6 is one of the key factors in the formation of gut tissue resident memory T cells from Naïve T cells
Source: PLoS Pathog. 2026 Mar 16;22(3):e1014052. doi: 10.1371/journal.ppat.1014052 (PMC13012526; doi:10.1371/journal.ppat.1014052)

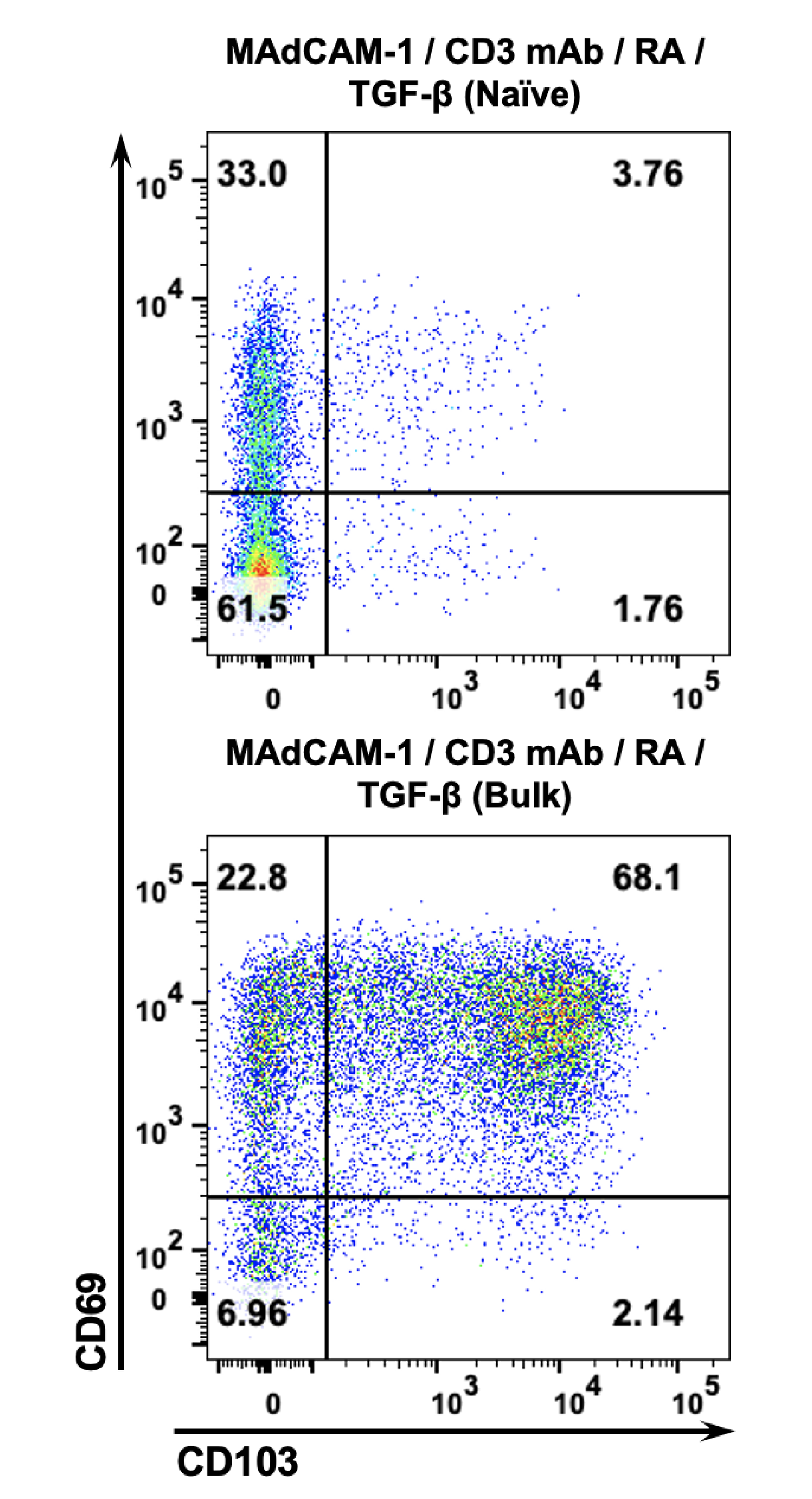

Supplement: S1 Fig — (A) Representative flow cytometry dot plots of PBMC derived naïve (upper) and bulk (lower) CD4+ T cells costimulated with MAdCAM-1 (+ RA and TGF-β). Y-axis indicates CD69, x-axis indicates CD103. Frequencies of CD69+/CD103+ cell as indicated. (TIF) [file ppat.1014052.s001.tif]

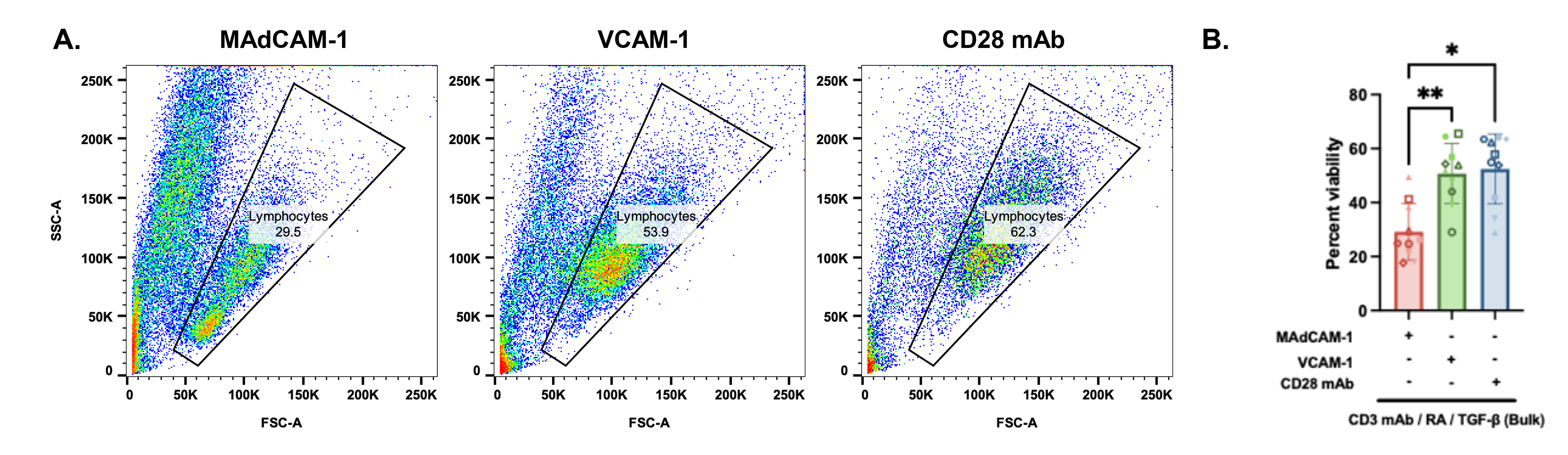

Supplement: S2 Fig — (A) Representative flow cytometry dot plots and lymphocyte gating of bulk CD4+ T cells that were costimulated with MAdCAM-1, VCAM-1 or CD28 mAb (+ RA and TGF-β). (B) Flow cytometric analysis of the lymphocyte gated population compared between the costimulatory ligands (n = 10). Error bars indicate standard deviation (*: p < 0.05, **: p < 0.01, ***: p < 0.001, ****: p < 0.0001). (TIF) [file ppat.1014052.s002.tif]

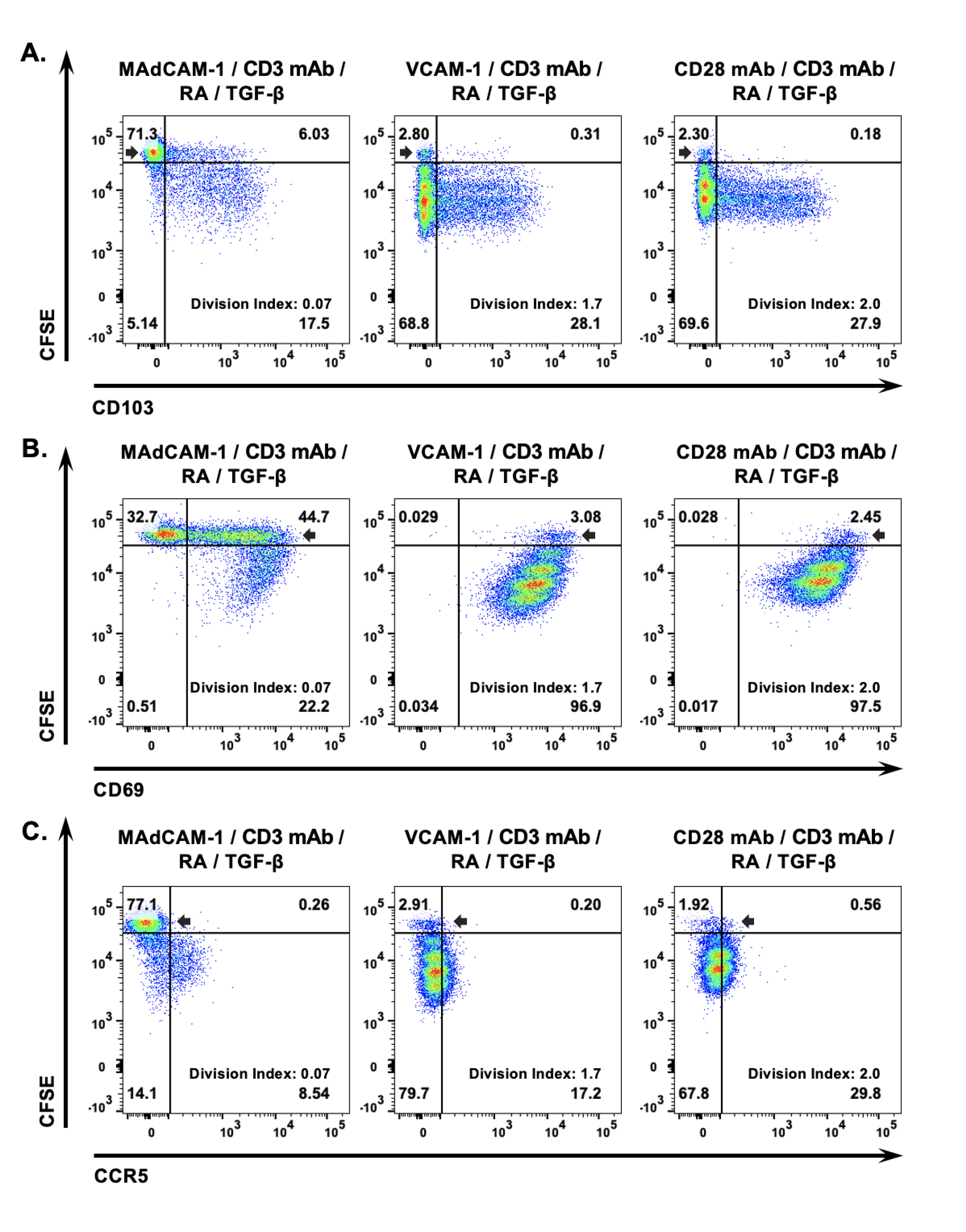

Supplement: S3 Fig — Flow cytometric dot plots from a representative donor naïve CD4+ T cell proliferation by dye dilution (y-axis) and (A) CD103, (B) CD69 and (C) CCR5 expression (x-axis) following MAdCAM-1 costimulation in the presence of RA and TGF-β. Division 0 (d0) is indicated by a black arrow. Division index as indicated. (TIF) [file ppat.1014052.s003.tif]

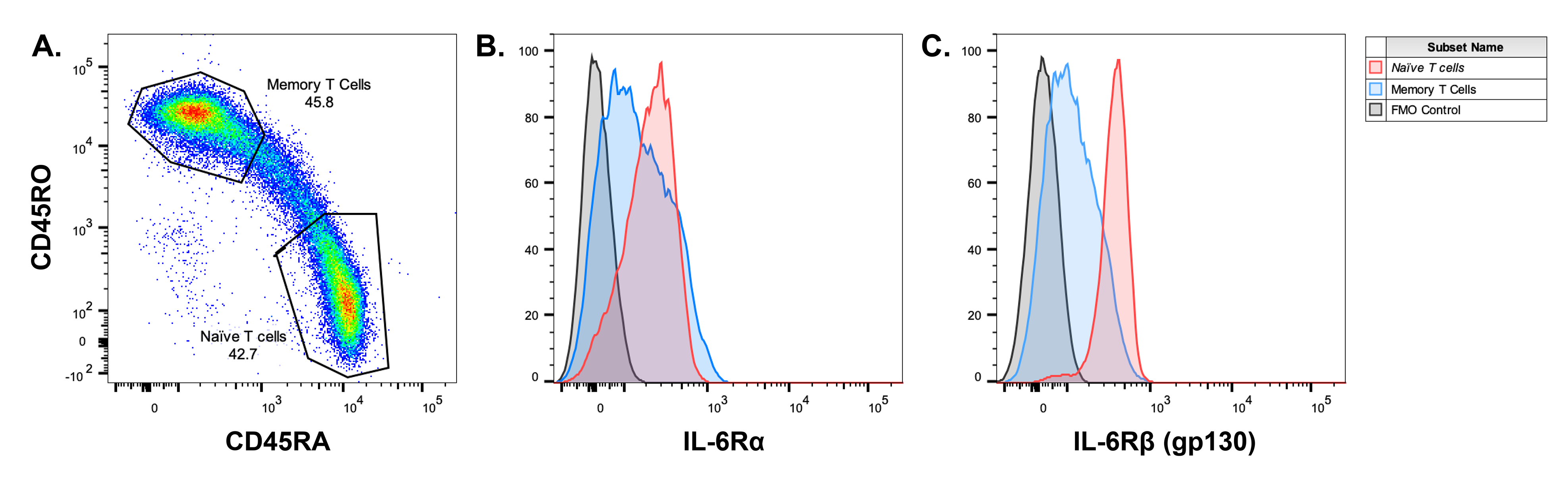

Supplement: S4 Fig — (A) CD45RO/CD45RA gating by flow cytometry from a representative donor. (B) IL-6Rα levels of each gated population. B) IL-6Rβ levels of each gated population. (TIF) [file ppat.1014052.s004.tif]

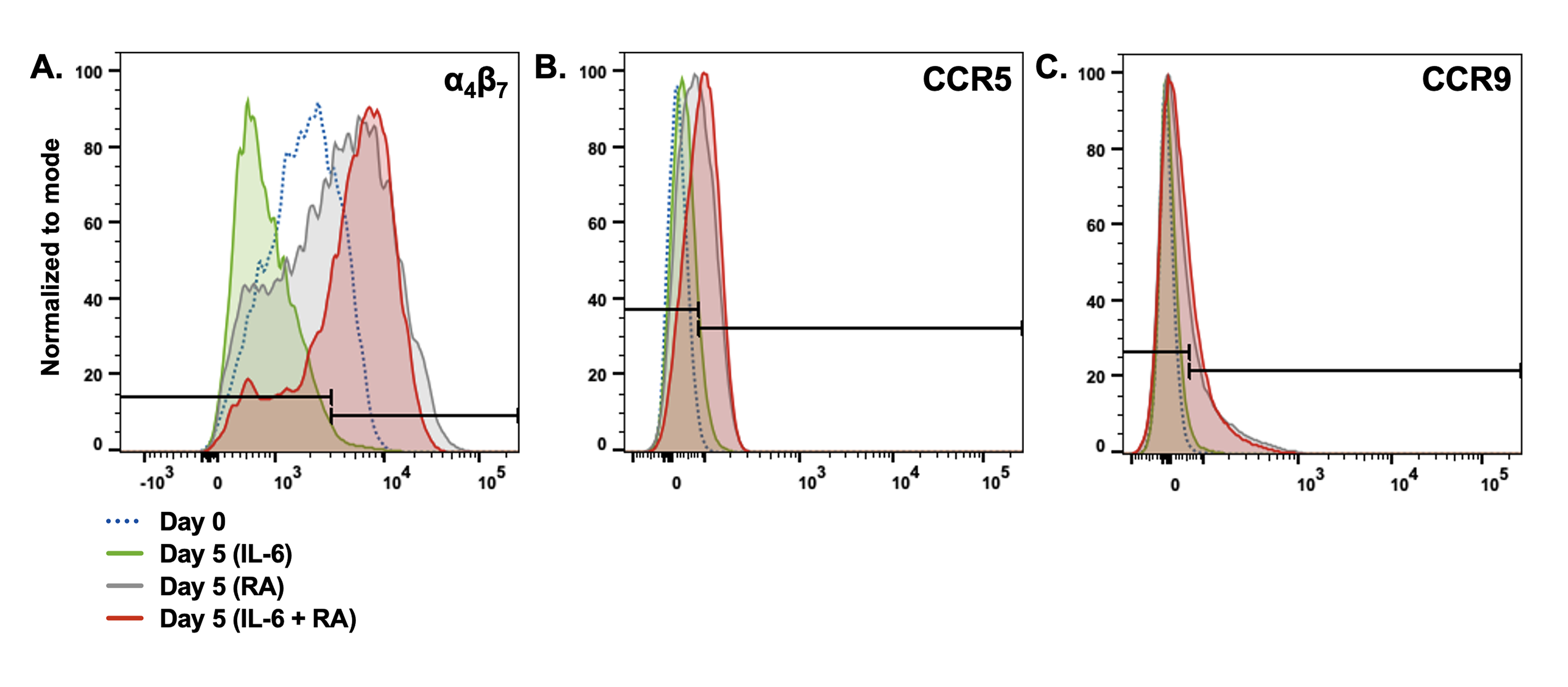

Supplement: S5 Fig — Measured proteins include (A) α4β7, (B) CCR5 and (C) CCR9. The gates used in Fig 3E are indicated. All plots are from the same representative donor. (TIF) [file ppat.1014052.s005.tif]

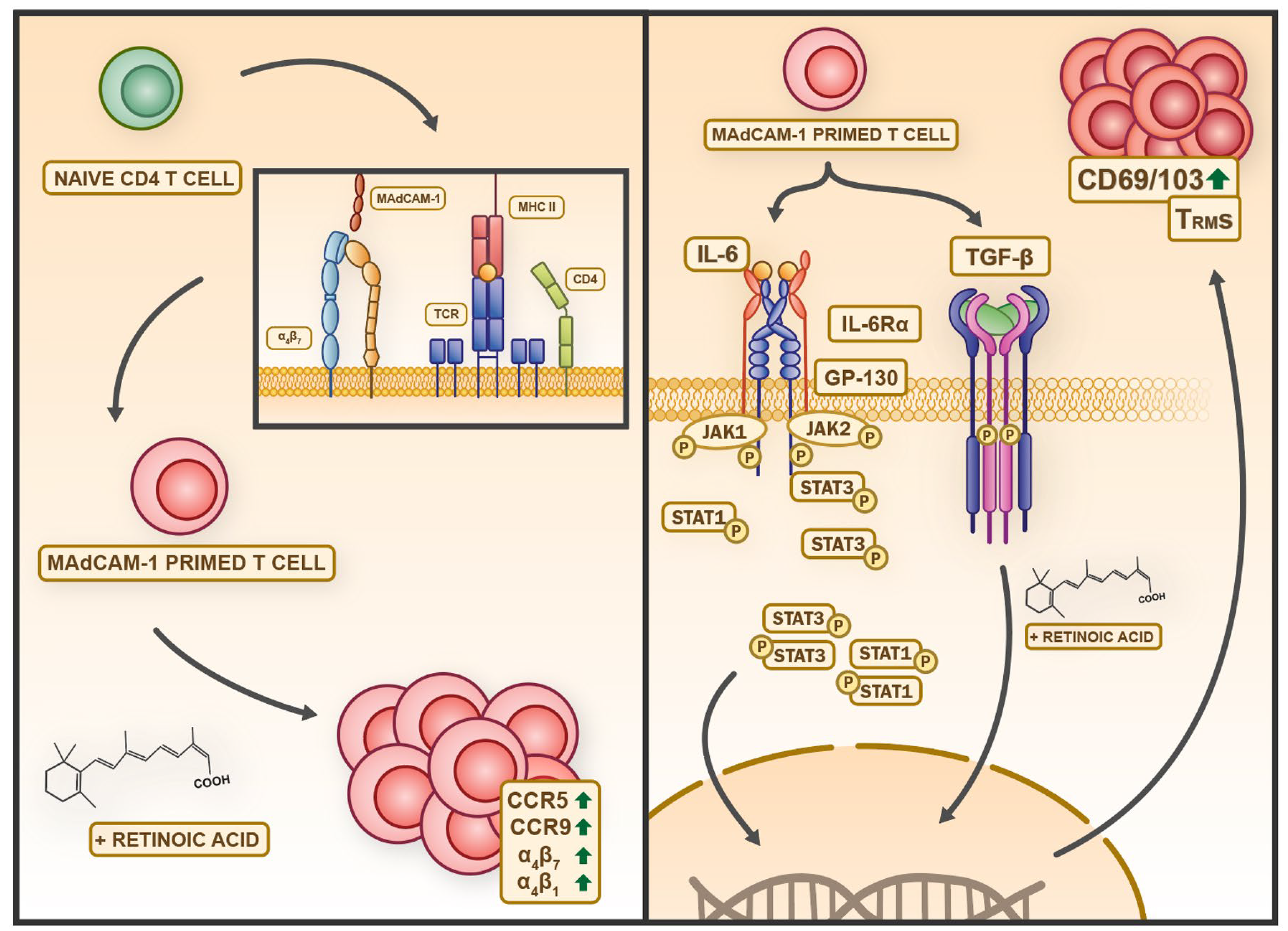

Supplement: S6 Fig — Schematic representation of the differentiation of naïve CD4 ⁺ T cells primed through engagement of α4β7 by MAdCAM-1. Integrin signaling in the presence of Retinoic acid (RA), TGF-β, and cytokine-driven activation of the JAK/STAT pathway promotes tissue-resident memory T cell (TRM) differentiation with upregulation of CD69 and CD103 (αEβ7). (TIF) [file ppat.1014052.s006.tif]
