# Supplementary material for: IL-6 is one of the key factors in the formation of gut tissue resident memory T cells from Naïve T cells
Source: PLoS Pathog. 2026 Mar 16;22(3):e1014052. doi: 10.1371/journal.ppat.1014052 (PMC13012526; doi:10.1371/journal.ppat.1014052)
Supplement: S1 Table — (PDF) [file ppat.1014052.s007.pdf]

Supplemental Table 1. List of antibodies used in the study.

Target	Fluorophore	Clone	Company	Cat. Number	
CD103	PE-cy7	Ber-ACT8	Biolegend	350212	FACS
CD69	BV480	FN50	BD	747519	FACS
CCR9	APC	L053E8	Biolegend	358908	FACS
CCR5	BV421	J418F1	Biolegend	359118	FACS
CD3	BV605	UCHT1	Biolegend	3004660	FACS
CD3	APC-H7	DK7	BD	560176	FACS
CD4	AF700	RPA-T4	Biolegend	IV T114	FACS
CD4	PE	RPA-T4	BD	561844	FACS
CD8	BB700	RPA-T8	BD	566452	FACS
IL-6Rα	PE	UV4	Biolegend	352806	FACS
IL-6Rβ	BB700	AM64	BD	746079	FACS
CD45RO	BV650	UCHL1	BD	748367	FACS
CD45RO	PE	UCHL1	BD	555493	FACS
CD45RA	APC-H7	HI100	BD	560674	FACS
CD45RA	FITC	HI100	BD	555488	FACS
β7	PE	FIB504	BD	555945	FACS
β7	APC	FIB504	BD	551082	FACS
β1 (CD29)	PE	MAR4	BD	555443	FACS
α4β7	FITC	ACT-1	In lab	-	FACS
STAT1	-	1/Stat1	BD	610116	WB
p-STAT1	-	4a	BD	612233	WB
p-STAT2	-		Millipore	07224	WB
STAT3	-	D1B2J	Cell Signal	30835	WB
p-STAT3	-	D3A7	Cell Signal	9145	WB
STAT5	-	D3N2B	Cell Signal	25656	WB
p-STAT5	-	D47E7	Cell Signal	4322	WB
Actin	-	AC-15	Santa Cruz	sc-69879	WB
