# Supplementary material for: IL-6 is one of the key factors in the formation of gut tissue resident memory T cells from Naïve T cells
Source: PLoS Pathog. 2026 Mar 16;22(3):e1014052. doi: 10.1371/journal.ppat.1014052 (PMC13012526; doi:10.1371/journal.ppat.1014052)
Supplement: Attachment — Submitted filename: Reviewers comments and rebuttal .pdf [file ppat.1014052.s008.pdf]

Reviewers' comments (if peer reviewed):

Reviewer's Responses to Questions

Part I - Summary

Please use this section to discuss strengths/weaknesses of study, novelty/significance, general execution and scholarship.

Reviewer #1: Here the authors have used in vitro differentiation techniques to explore putative signals important for imprinting a “gut homing” phenotype to differentiating naïve CD4 T cells. The authors explore use of retinoic acid, TGFb, +/- IL6. The authors find that addition of IL6 increases the likeliness of naïve CD4 T cells differentiating into a CD4 T cell with a “gut-homing” phenotype.

Reviewer #2: In this paper the authors explore the different factors that can mediate differentiation of naive CD4T cells into GI TRM phenotype like with the ultimate goal to help understand what intervention can help restore of increase TRM CD4 T cells in the GI which is known to help with gut mucosa integrity and lower inflammation.

The authors have previously shown the involvement of MAdCAM-1 (when combined with RA) into priming CD4T cells to express TRM markers without knowing the origin of this differentiation (naïve or memory cells). This time they are exploring the importance of naïve and memory presence and ratio and are exploring other factors involved in the process.

The main findings of this study are: In PBMCs of healthy donors, MAdCam-1 +RA prime naïve CD4+T cells to express GI TRM cell markers and that IL-6 and TGFb promote this differentiation. They are also clearly showing and stating numerous times that other pathways, yet to be identified, are involved in the process.

Thanks for this hard work and submission.

Reviewer #3: In this manuscript, Kim et al al investigates te factors that mediate differentiation of naïve CD4+ T cells into cells presenting a gut tissue resident memory CD4+ T cell phenotype. TRMs populate mucosal sites and play a critical role in local immune responses. Gut TRM cells persist for extended periods in the gut mucosa where they rapidly respond to invading pathogens and provide long lasting protection. They applied a combination of stimuli to naïve CD4+T cells in order to prime them to adopt a TRM phenotype. They report that, in order to fully differentiate into TRMs an additional soluble factor provided by memory T cells was required. They identified IL-6 as a key factor that induces the expression of TRM -associated markers, including CD69, CD103 and CCR5. This unique combination of stimuli promoted TRM differentiation despite low level proliferation. TRM differentiation was mediated through JAK/STAT signaling, and antagonists that target JAK/STAT pathways suppressed MAdCAM-1 mediated TRM cell formation. They report that MAdCAM-1 works together with TGF-β, RA and IL-6 in this process. The study is important because such information may aid in the design of next generation adjuvants and effective mucosal vaccines. Additionally, each of these factors may be targeted to treat excessive gut inflammation associated with conditions like inflammatory bowel disease or HIV infection. **The manuscript is interesting, well written and of potentially high impact. The work is well designed and carefully carried out.**

Part II – Major Issues: Key Experiments Required for Acceptance
Please use this section to detail the key new experiments or modifications of existing experiments that should be absolutely required to validate study conclusions.

Generally, there should be no more than 3 such required experiments or major modifications for a "Major Revision" recommendation. If more than 3 experiments are necessary to validate the study conclusions, then you are encouraged to recommend "Reject".

Reviewer #1: 1. The in vivo relevance of the findings are completely unclear. The authors should compare transcriptional profiles of IL-6-differentiated naïve CD4 T cells to mucosal CD4 T cells taken ex vivo (biopsies, or do similar experiments w/ NHPs).
2. The biological relevance is unclear. Are there longitudinal decreases in gut homing CD4 T cells in patients treated with Tocilizumab?
3. In inflamed individuals, are there associations between the levels of plasma IL6 and the frequency of memory CD4 T cells with a gut homing phenotype?
4. The authors also use ruxo to decrease signaling through JAK/STAT. The NIH clinical center is full of patients who have been treated with this drug. What happens to the frequencies of gut-homing phenotype+ CD4 T cells pre/post?

Reviewer #2: I have a few comments/suggestions that I hope can help to improve the manuscript.

- The origin of the TRM CD4T cell in the GI is still a controversial topic. A nice review that might need a little update (PMID: 30897205) is reporting on this topic and summarize studies into the origins of CD4+ memory T-cell populations and discuss studies addressing CD4+ TRM differentiation in barrier tissues. The authors might want to refer to it as it reports on a lot of the data the authors are looking into.
https://pmc.ncbi.nlm.nih.gov/articles/PMC6459775/pdf/IMM-157-3.pdf (PMID: 30897205)Nguyen

The reviewer's suggestion to include this review (Nguyen et al.) has significantly improved our manuscript's introduction as it covers reports framing our study in a more comprehensive way.

- IL-6 has been previously reported in numerous manuscripts as a pro-inflammatory and protective factor in the GI. As this Interleukin is the focus of this manuscript, I was surprised to not see any summary on what has been previously published in the introduction. IL-6 benefits in GI inflammation is not new.

This reviewer's comment is well taken and we added three references, including the review suggested, and a paragraph in the introduction (Nguyen et al., Waldner et al., Pawlowska-Kamieniak et al.).

- An important study design challenge that the authors are reporting is about the ratio of naïve versus memory cells in their cultures as this is playing an important role in the differentiation of TRMCD4+T cells. I am curious to know why the authors didn't look at the ratio of those sub-population of cells across all donors (Before culture) and tested the same ratio in vitro if they wanted to be as close as possible to an in vivo condition.

We apologize we did not make clear in our first submission that the ratio of naïve vs memory is tested prior to cell culture (we have clarified this in the text). In our previous study we employed only bulk CD4+ T cells and noticed a variability among donors CD4+ T cells in producing TRMs when stimulated with MAdCAM-1. In this study we conducted both a pre-culture screening of the naïve vs memory ratio in bulk cultures, while also isolating memory and naïve CD4+ T cells separately from PBMCs to directly clarify the correlation between TRMs generation and naïve/memory CD4+ T cells frequencies (see also comment below).

- Did the authors look at the presence of IL6 Receptor on naïve and memory and TRM CD4+T cells?

Following the reviewer's comment we added a supplementary figure (SFig.3) to the manuscript of an experiment showing IL6R expression by flow cytometry, and determined that IL6R is expressed on most CD4+ T cells

- One major missing data in the different culture experiences is the differential between cell input, cell dying and cells expanding.

Following this reviewer's comment, we have provided data below on cell expansion and cell death using a representative donor:

- *MAdCAM-1 has the highest proportion of debris/dead cells ~75%*
- *VCAM-1 has a lower proportion of debris/dead cells ~50%*
- *As one would expect, when debris/dead cells are gated out, you end up with mostly alive cells in these cultures (assessed by L/D staining)*

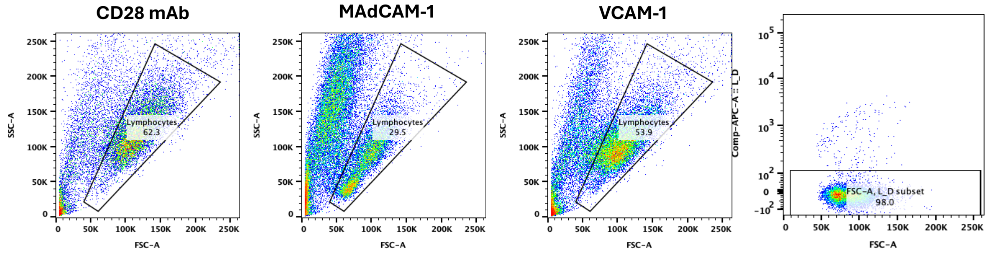

- The number of donors and how many were used for each experiment is confusing. Figure 1 A report data on 43 donors, but then figure1C has 8 and figure 2 B has 4 but then Figure 2D has a n=3 but this is the repeats? how many donors? Figure 4D has 4D was done on 11 donors and figure4 E on 6 donors. I am of course not expecting the authors to run all experiments on all 43 donors, but can those numbers be justified and how those donors were selected for each steps need to be included or why other were excluded and importantly are they the same donors across the manuscript out of the initial 43. Sorry if I missed these information.

- *We reanalyzed the data collected on bulk CD4 cells from a previous study. We noticed a trend in the frequencies of naïve T cells and the efficiency of TRM formation. This prompted us to analyze additional samples to see if the trend held. This resulted in new information on a total of 43 bulk CD4 T cell cultures from independent donors included in figure 1. At the same time, with these new samples we carried out additional analyses including isolation of memory and naïve subsets, and inhibition with several drugs. That is why the number of samples in all the figures that followed included a smaller number of samples than in figure 1.*
  For example, we tested ruxolitinib and AZD1480 inhibition with 11 different donors (figure 4D). However, we were limited in the number of cells we obtained from each donor, so not all donors could be tested for both ruxolitinib and AZD1480 (figure 4E), which was analyzed with 6 donors.

  - The authors have collected supernatant from their culture, did you conduct a full characterization of it to better identify other factors that might be involved in the differentiation of the TRM.

Because of limited funds we were unable to characterize all potential factors in the supernatant. Instead, we took a hypothesis driven approach and tested additional factors (IL-23 and IL-35) implicated in Th17 and Treg differentiation which are both potentially relevant subsets for GALT tissue resident populations. Both factors did not induce TRM differentiation. Because this data was negative, we decided not to include it in the interest of brevity.

Reviewer #3: (No Response)

Part III – Minor Issues: Editorial and Data Presentation Modifications
Please use this section for editorial suggestions as well as relatively minor modifications of

existing data that would enhance clarity.

Reviewer #1: (No Response)
Reviewer #2: Minor comments:
Figure1C: missing days of culture.
And number of donors for each panel on each figure legend need to be indicated and differentiated if this is a repeat or individual donors.

All experiments for figure 1 were cultured to Day 7 and we modified the figure legend accordingly. We have also specified the number of donors for each panel on each figure.

A small suggestion about the title I think "differentiation" instead of "Formation" might be more accurate- and IL-6 is "ONE" of the key factor would be more accurate.

We changed the manuscript's title as this reviewer suggested.

Reviewer #3: There were a few things that I found lacking:
• The gating and sorting are not straightforward. They provide many gating strategies in their figures but don't detail how T cell subsets were isolated and validated. For example, they say purity of CD4+ T cell isolation was determined by flow cytometry but don't mention if subsets were also determined and what the final percentages were.

> *T cell subsets were isolated using stem cell technologies magnetic beads for bulk CD4+ T cells with EasySep Human CD4+T cell enrichment and naïve CD4+ T cells with EasySep Human Naïve CD4+ T cell isolation kit. We then assessed naïve/memory frequencies by flow cytometry using CD45RA/CD45RO staining.*
>
> *Our gating strategies can be seen in figure 1B. CD45RO T cells present two peaks of low vs high expression. The two populations were gated at the center between the high vs low peaks.*

• Related to this, they pretty loosely use "memory subset" and allude to memory subsets in ways that are confusing/ambiguous. It is not clear to me where they determine different memory subsets; I assume they are using memory subset only in contrast to naïve T cells. Similarly, they reference effector memory cells but don't actually look specifically at them. It's not clear if/how these findings apply to central memory cells. This is especially relevant because they discuss viral reservoirs in the discussion.

This reviewer is correct as we mainly use CD45RO+ to characterize memory T cells which is an imperfect marker for memory cells. We have now clarified in the results section of the text our use of CD45RO as a marker for memory and its limitations. Regarding memory cell subsets (effector vs central), the reviewer's comment is valid. We are very much interested in determining which memory subset (central vs. effector) is mainly responsible for

secreting factors conditioning naïve cells to differentiate into TRMs. However, this study was done on primary cells which constrained us to limit our ambition to answering the question regarding the specific factors relevant to TRM formation. We added a sentence at the beginning of the results to clarify this issue.

• Figure 1a is confusing. The parts that reference it in the results are unclear. The use of a second degree polynomial equation does not make sense to me, since they are testing two unrelated experimental conditions. The r values are so low, I think claiming there's actually a correlation is disingenuous.

This reviewer is correct. We agree that it is better to separate the two experimental conditions (bulk vs. naïve CD4 T cells) from figure 1A. We have modified figure 1A by eliminating the purified naïve CD4+ T cell data and left the ex vivo naïve/memory frequencies data.

• Related to figure 1, it's not clear to me whether the ratio of naïve to memory cells was just due to the relative ratio from the donors or whether it was artificially manipulated. If it is due to differences in donors, there may be other differences (age, pathogens, microbiome etc) that may be contributing to the differences in relative ratios, which is not stated as a limitation. They go back to validate their findings experimentally so this isn't a big deal in the overall scheme of the study but worth clarifying.

We included both ex-vivo ratio and manipulated ratio. We apologize we did not make clear in our first submission that the ratio was tested before culture. In our previous study we employed only bulk CD4+ T cells and noticed a variability among donors CD4+ T cells in producing TRMs when stimulated with MAdCAM-1. In this study we conducted both a pre-culture screening of the naïve vs memory ratio in bulk cultures, while isolating memory and naïve CD4+ T cells separately from PBMCs to directly clarify the correlation between TRMs generation and naïve/memory CD4+ T cells frequencies. We now added more clearly in the text that the frequencies were determined before culture was initiated.

• Several claims in the discussion are not cited and do not resonate with how I understand the differentiation of Trms, which makes me question them. I would also recommend that they go back to previous research to better contextualize these new findings.

Thanks to this reviewer's comments we have modified and improved the manuscript. We added more references for both the introduction and discussion.

Overall, I think this study is important, as previous work targeting a4b7 with monoclonal antibodies has led to inconsistent results. By examining additional factors and finding new targets downstream of MAdCAM1 signaling, they may be able to better target dysregulation in the differentiation of Trms during HIV.

Although the main emphasis of this study is on TRMs ontogeny, these authors are well aware of the inconsistencies of previous work on targeting a4b7 in the context of HIV-1 infection. Hopefully, this study may open a new aspect that may help better understand the role of a4b7 in HIV-1 infection.

PLOS authors have the option to publish the peer review history of their article (what does this mean?). If published, this will include your full peer review and any attached files.

If you choose "no", your identity will remain anonymous but your review may still be made public.

Do you want your identity to be public for this peer review? For information about this choice, including consent withdrawal, please see our Privacy Policy.
Reviewer #1: No
Reviewer #2: No
Reviewer #3: No

[NOTE: If reviewer comments were submitted as an attachment file, they will be attached to this email and accessible via the submission site. Please log into your account, locate the manuscript record, and check for the action link "View Attachments". If this link does not appear, there are no attachment files.]
