# Supplementary material for: IL-6 is one of the key factors in the formation of gut tissue resident memory T cells from Naïve T cells
Source: PLoS Pathog. 2026 Mar 16;22(3):e1014052. doi: 10.1371/journal.ppat.1014052 (PMC13012526; doi:10.1371/journal.ppat.1014052)

DEPARTMENT OF HEALTH & HUMAN SERVICES

Public Health Service

National Institutes of Health
Bethesda, Maryland 20892

02/11/2026

Dear Editors,

We are grateful for the time and effort dedicated to consider publication of our manuscript entitled *" IL-6 is one of the Key Factors in the Formation of Gut Tissue Resident Memory T Cells from Naïve T cells "* The insightful and constructive comments have helped us improve the quality and clarity of our work.

In the revised version, we have carefully addressed all the comments and suggestions provided. Below, we detail our responses to each point raised, indicating where changes have been made in the manuscript. All revisions are clearly highlighted in the text.

1. *Could you test blocking IL-6 and IFN-β together when using supernatants?*

The reviewer raises a relevant issue. We note in the limitations section the following point:
"*Our results, as described above, indicate that factors other than IL-6 (e.g. IFN-β) can also promote T_{RM} cell differentiation, and we speculate that these factors promote the differentiation of distinct T_{RM} subsets. However, we have not yet identified the full complement of these factors and this represents a limitation of this study*."
Blocking both IL-6 and IFN-β would address this issue, at least in part. Unfortunately, the closure of our lab severely limited our ability to perform experiments, and we were only able to carry out new experiments in response to point #3. Hopefully we can find an opportunity to pursue this subject further.

—

2. *I would have liked the data on viability to be included in a supplementary figure or table. That way, readers can have a full appreciation of what is going on.*

As per the reviewer's request, we have added a new Supplementary Figure 2, which includes data on cell viability for each costimulatory signaling condition employed in the study. In addition, a sentence has been added to the first paragraph of the results section to reflect the inclusion of this new figure, as indicated by underlined text in the manuscript.

—

3. *Supplementary Figure 3 does not discriminate between naïve and memory CD4 T cells. It is unclear what is meant by CD4^dim CD3^dim, as this is not discussed elsewhere in the manuscript. However, from the flow plot, it appears that almost all CD4 T cells express IL-6R.*

The reviewer's point is well taken. To address this concern, we performed a new flow cytometry experiment to distinguish IL-6R expression on naïve versus memory T cells. That date can be. Found in the new Supplementary figure 4. We note in the third paragraph of the results section the following

observation: *CD4$^+$ CD45RA$^+$ naïve T cells also express higher levels of both IL6 receptor (IL-6Rα) and the signal transducer gp130 (IL-6Rβ) when compared to CD45RO$^+$ memory T cells (Supplemental Fig. 4).*

—

4. *"All study participants received written informed consent." Provided instead of received?*

This was a typo which has now been corrected in the revised version of the manuscript.

We believe that these revisions have significantly strengthened the manuscript, and we hope that it now meets the *PloS Pathogens* standards for publication.

Sincerely,

Claudia Cicala Ph.D.

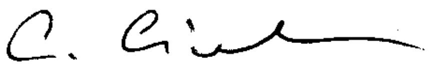

Supplement: Attachment — Submitted filename: Kim et al. Response to Reviewers .pdf [file ppat.1014052.s009.pdf]
